# Ferroptosis contributes to boar sperm deterioration during liquid semen storage

**DOI:** 10.3389/fvets.2026.1816003

**Published:** 2026-04-22

**Authors:** Mubashrah Mahmood, Tyler Weide, Karl Kerns

**Affiliations:** 1 Department of Animal Science, Iowa State University, Ames, IA, United States; 2 Department of Theriogenology, University of Agriculture, Faisalabad, Punjab, Pakistan; 3 Interdepartmental Genetics and Genomics, Iowa State University, Ames, IA, United States

**Keywords:** boar sperm, ferroptosis, lipid peroxidation, liquid storage, regulated cell death, spectral flow cytometry

## Abstract

Ferroptosis is an iron-dependent form of regulated cell death characterized by labile iron accumulation, glutathione depletion, mitochondrial dysfunction, and lipid peroxidation. Although ferroptosis has been linked to impaired spermatogenesis and male infertility *in vivo*, its occurrence and functional relevance in ejaculated spermatozoa during liquid semen storage at 17 °C has not been established. This study evaluated how ferroptosis inducers and inhibitors modulate boar sperm function during liquid semen storage at 17 °C and determined whether ferroptosis contributes to spontaneous sperm deterioration over time. Semen samples from five Duroc boars were treated with ferroptosis inducers: Erastin (system Xc^−^ antagonist) and RSL-3 (GPX4 inhibitor), inhibitors: Ferrostatin-1 and N-acetylcysteine (NAC), and vehicle/untreated controls. Sperm motility was assessed by computer-aided sperm analysis, and comprehensive ferroptosis-associated biomarkers were quantified using spectral flow cytometry with a 7-fluorophore panel including sperm identification (Hoechst 33342), labile ferrous iron (FerroOrange), glutathione (monochlorobimane), reactive oxygen species (DCFDA), lipid peroxidation (BODIPY 581/591 C11), mitochondrial membrane potential (MitoTracker Deep Red), and plasma membrane integrity (propidium iodide). RSL-3 induced declines in total and progressive motility accompanied by elevated Fe^2+^ accumulation, glutathione depletion, mitochondrial depolarization, and membrane disruption by day 3 (*p* < 0.05). Erastin produced similar but delayed effects, reaching maximum ferroptotic markers by day 7. Both Ferrostatin-1 and NAC mitigated oxidative injury and preserved motility and cellular integrity relative to inducers. Critically, untreated controls exhibited time-dependent increases in all ferroptosis-associated biomarkers over 7 days of liquid storage, indicating spontaneous ferroptotic activity independent of chemical induction. These findings establish ferroptosis as a mechanistically relevant contributor to sperm.

## Introduction

Cell death is a key regulator of normal biological function and is regulated by multiple processes (1). Historically, programmed cell death was understood primarily through apoptosis; however, recent research has revealed multiple regulated cell death (RCD) pathways, including necroptosis, pyroptosis, and ferroptosis (2–4). Accidental cell death, or necrosis, occurs due to chemical, mechanical, or physical trauma to cells, whereas regulated cell death is governed by precise interactions among specific proteins and genes (1). Ferroptosis, discovered only in 2012, represents a novel nonapoptotic form of RCD characterized by iron-dependent lipid peroxidation distinct from apoptotic mechanisms (5, 6). Ferroptosis occurs through labile iron-catalyzed Fenton reactions that generate reactive oxygen species (ROS), leading to overwhelming lipid peroxidation when antioxidant defenses are compromised (5–7).

Normal cellular metabolism of oxygen generates reactive oxygen species (ROS), which play dual roles in sperm biology (8, 9). ROS have been detected in human spermatozoa since 1943 and at physiological concentrations are necessary for normal spermatogonial function, sperm capacitation, and fertilization (8, 9). However, excess ROS generation causes cellular damage, defective sperm function, lipid peroxidation, plasma membrane disruption, protein dysfunction, and DNA fragmentation in humans (10, 11) and across mammalian species (12), ultimately compromising sperm viability and male fertility (9, 13). ROS-induced oxidative damage particularly affects spermatozoa because sperm plasma membranes are lipid-rich, contain limited cytoplasmic antioxidant defenses, and depend heavily on mitochondrial energy production, creating an ideal environment for oxidative injury during extended storage (14, 15). In boar spermatozoa specifically, liquid semen storage at 17 °C leads to progressive ROS accumulation and oxidative stress that correlates with declining sperm quality (16, 17).

Intracellular iron balance is critical for normal spermatogenesis, but iron overload can trigger ferroptosis in germ cells, impairing normal sperm development (18). Since ferroptosis was first described, accumulating evidence suggests ferroptosis-related mechanisms contribute to male infertility (18–23). Ferroptosis has been implicated in asthenozoospermia (reduced sperm motility) through promotion of lipid peroxidation and mitochondrial dysfunction (24). Age-related fertility decline in males involves ROS-triggered ferroptosis in testicular tissue (23). Environmental pollutants such as PM₂.₅ disrupt spermatogenesis by triggering testicular ferroptosis (20) through iron accumulation, glutathione depletion, and GPX4 suppression, ultimately affecting sperm count and fertility. Clinical evidence from asthenozoospermic men demonstrates reduced SLC7A11 and GPX4 expression consistent with ferroptotic activation (7, 25). Recent evidence demonstrates ferroptosis involvement in sperm cryoinjury, with studies in goats and rams showing that ferroptosis inhibitors (Liproxstatin-1, Ferrostatin-1, deferoxamine) improve post-thaw sperm motility, mitochondrial function, membrane integrity, and DNA integrity (16, 26, 27).

Despite evidence for ferroptosis in testicular tissue and cryopreservation, ferroptosis in ejaculated boar spermatozoa during routine liquid semen storage has not been directly evaluated. In the swine industry, >95% of pig production relies on artificial insemination with liquid-stored semen, typically maintained at 15–17 °C for 3–7 days before insemination (28). Understanding whether ferroptosis contributes to sperm viability loss during liquid storage has substantial implications for improving semen extender formulations and enhancing post-storage fertility outcomes. Furthermore, ferroptosis represents a distinct RCD pathway potentially amenable to pharmacological targeting, unlike constitutive oxidative damage. Therefore, this study aimed to determine whether ferroptosis can be experimentally induced in boar sperm, whether ferroptosis emerges spontaneously during liquid semen storage, and whether ferroptosis inhibitors can preserve sperm function. We hypothesized that ferroptosis can be induced in boar sperm cells and that the sperm deterioration observed during liquid semen storage is mediated, at least in part, by ferroptosis.

## Materials and methods

### Experimental design

Semen samples from sexually mature Duroc boars were analyzed. Ferroptosis inducers (Erastin and RSL-3), ferroptosis inhibitors (Ferrostatin-1 and N-acetylcysteine), and vehicle controls (0.1 and 0.2% DMSO) were used as treatments and controls (Table 1). Each biological replicate received all treatments, vehicle controls, and untreated controls. Sperm motility was analyzed using computer-aided sperm analysis (CASA). The experimental design included both ferroptosis modulation trials and evaluation of spontaneous ferroptosis during liquid storage.

**Table 1:** Experimental treatment groups.

Sample No.	Treatment group	Description	Vehicle composition (%)
1	Sample only	Control	–
2	Sample + DMSO (1X)	Vehicle 1X	0.1%
3	Sample + Erastin (1X)	Ferroptosis Class I Inducer 1X	0.1%
4	Sample + RSL-3 (1X)	Ferroptosis Class II Inducer 1X	0.1%
5	Sample + DMSO (2X)	Vehicle 2X	0.2%
6	Sample + Ferr-1 (2X)	Ferroptosis Inhibitor 2X	0.2%
7	Sample + NAC (2X)	Ferroptosis Inhibitor 2X	0.2%

Semen samples were divided into seven treatment groups to assess ferroptosis induction and inhibition during storage. Erastin and RSL-3 served as ferroptosis inducers, while Ferrostatin-1 (Ferr-1) and N-acetylcysteine (NAC) acted as inhibitors. DMSO (0.1% or 0.2%) was used as the vehicle control to maintain uniform solvent exposure across treatments.

Pilot studies using image-based flow cytometry (IBFC) were performed on semen from six boars at days 0, 3, and 7 to screen candidate ferroptosis biomarkers and optimize fluorophore combinations for single-cell analysis (Supplementary Tables S1, S2). These exploratory experiments were designed to identify temporal patterns of iron accumulation, glutathione depletion, oxidative stress, and mitochondrial depolarization, and to refine gating strategies (Supplementary Figures S1, S2). Based on these pilot findings, day 3 was identified as a critical time point at which coordinated ferroptosis-like changes became apparent (Supplementary Figure S3). Because the pilot IBFC panels were optimized for biomarker screening and spatial visualization rather than simultaneous high-dimensional quantification, full multiparametric validation required development of an integrated spectral flow cytometry (SFC) assay.

Spectral flow cytometry validation was therefore conducted on semen from five boars at days 0, 1, 3, and 7 to quantitatively confirm ferroptosis induction, inhibition, and spontaneous progression in untreated semen using a finalized 7-fluorophore panel (Supplementary Table S3). Full-spectrum detection and unmixing enabled simultaneous resolution of fluorophores with partially overlapping emission spectra, improving discrimination of labile iron, glutathione status, peroxide-sensitive oxidative stress, lipid peroxidation, mitochondrial membrane potential, and plasma membrane integrity within the same cellular event under standardized acquisition conditions. This integrated approach allowed coordinated assessment of defining ferroptosis hallmarks and downstream functional consequences in a single analytical framework.

### Semen collection

Six sexually mature commercial Duroc boars were used for IBFC pilot studies, and five boars were used for SFC validation studies. Semen samples were collected using the standard two-gloved hand procedure and extended within 10 min of collection using Preserve Xtreme semen extender to a final concentration of 40 million sperm/mL. Extender temperature was maintained within 2 °C of the semen sample temperature. Extended samples were transported to Iowa State University on the same day and stored at 17 °C.

### Sperm motility evaluation

Sperm motility was assessed using AndroVision (CASA; Minitube, Germany) at 10x magnification. Semen samples were mixed thoroughly to ensure even sperm distribution, and 20 μL of each sample was prewarmed in a 0.75 mL microcentrifuge tube using a Fisherbrand Isotemp warm plate to 37 °C for 15 min. After gentle mixing, 3.5 μL was transferred to a prewarmed glass disposable counting chamber (20 μm, Minitube) using a micropipette. The chamber was immediately analyzed using a Zeiss Axioscope 5 microscope (Carl Zeiss Microscopy, LLC) equipped with a Basler ac2440-75uc camera (Basler AG) and 10×/0.25 A-Plan objective lens using Minitube AndroVision software. Four microscopic fields totaling >600 sperm cells were analyzed to determine total motility (TM) and progressive motility (PM). Only ejaculates with >80% TM were selected for study. Samples were incubated with treatments immediately after motility assessment.

### Reagents and media

Erastin (Cat. No. HY-15763; MedChemExpress LLC, New Jersey, United States), RSL-3 (Cat. No. 192881; Cayman Chemical, Ann Arbor, MI, United States), Ferrostatin-1 (Cat. No. A4371-5 mg; APExBIO Technology LLC, Houston, TX, United States), and N-acetyl-L-cysteine (Cat. No. AAA1540914; Thermo Fisher Scientific Chemicals, Ward Hill, MA, United States) were dissolved in dimethyl sulfoxide (DMSO; Cat. No. BP231-100; Fisher BioReagents, Fair Lawn, NJ, United States) at stock concentrations detailed in Table 1. Fluorescent probes included FerroOrange (Cat. No. F374; Dojindo Laboratories, Kumamoto, Japan), Hoechst 33342 (Cat. No. 33342; Invitrogen, Waltham, MA, United States), 6-carboxy-2′,7′-dichlorodihydrofluorescein diacetate (Carboxy-DCFDA; Cat. No. C369; Invitrogen, Waltham, MA, United States), BODIPY 581/591 C11 (Cat. No. D3861; Invitrogen, Waltham, MA, United States), MitoTracker Deep Red FM (Cat. No. M22426; Invitrogen, Waltham, MA, United States), monochlorobimane (MCB; Cat. No. 69899-5MG; Sigma-Aldrich, Burlington, MA, United States), and propidium iodide (PI; Cat. No. P1304MP; Thermo Fisher Scientific Chemicals, Ward Hill, MA, United States). Preserve Xtreme semen extender was obtained from GENEPRO (Madison, WI, United States).

Fluorophores were reconstituted according to the manufacturer’s instructions in a dark room to prevent bleaching. Detailed reconstitution procedures are provided in Supplementary Methods. Image-based flow cytometry (IBFC) biomarker panels used in the pilot study are detailed in Supplementary Tables S1, S2. SFC gating strategies are shown in Supplementary Figures S1, S2. Representative biomarker fluorescence localization in sperm cells is shown in Supplementary Figure S6.

### Spectral flow cytometry analysis

The BD FACSDiscover A8 SFC (Beckman Coulter, United States), equipped with five lasers (355, 405, 488, 561, and 638 nm) and a 78-channel full-spectrum detector array, was used for all analysis. Ten million sperm cells were stained at each time point for each treatment. Samples were centrifuged at 110 × g for 4 min, supernatants removed, and sperm pellets resuspended in 200 μL porcine non-capacitation media [modified TL-HEPES without calcium and bicarbonate (29)] containing the seven fluorophores listed in Supplementary Table S3. Multiparametric flow cytometric approaches have proven valuable for comprehensive sperm quality assessment across species (30, 31). Final fluorophore concentrations were: FerroOrange 5 μM, propidium iodide 0.25 μM, Hoechst 33342 1.8 μM, Carboxy-DCFDA 0.5 μM, BODIPY C11 50 nM, and MitoTracker Deep Red 0.5 μM. Samples were incubated at 37 °C for 30 min in the dark, then centrifuged again, supernatants removed, and pellets resuspended in 120 μL phosphate-buffered saline. Hundred microliters of stained samples were transferred to 96-well U-bottomed microplates for spectral data acquisition using BD FACSDiscover A8 Software with standardized laser power and flow settings. Single-stained.

### Spectral flow cytometry data analysis

FCS files were analyzed using FlowJo v10 (BD Biosciences). Hoechst 33342 fluorescence was plotted against forward scatter area (FSC-A) to identify nucleated sperm and exclude debris (Supplementary Figure S1A). FSC-A versus FSC-H was used to select single sperm cells and exclude aggregates (Supplementary Figure S1B). Single sperm populations were analyzed for propidium iodide, MitoTracker Deep Red, monochlorobimane (MCB), Carboxy-DCFDA, BODIPY C11, and FerroOrange to quantify functional and oxidative biomarkers (Supplementary Figures S1C–H). The FerroOrange-positive sperm population (FO+), representing candidate ferroptotic cells based on labile iron accumulation, was subsequently analyzed for all other biomarkers to characterize ferroptosis progression within iron-accumulating cells (Supplementary Figures S2D–G). Positive and negative populations were defined for each fluorophore using unstained and single-stained controls, and frequency of positive cells (% of parent and grandparent population) was exported as comma-separated files for statistical analysis.

### Statistical analysis

Flow cytometry-derived percentage data were analyzed using Minitab (version 21; Minitab LLC, PA, United States). One-way ANOVA with Tukey’s *post hoc* test was used for single time-point comparisons to assess treatment effects. For the untreated control, separate one-way ANOVA with Tukey’s *post hoc* test evaluated time-dependent changes across storage days. Results are reported as mean ± SEM with significance set at *p* < 0.05.

## Results

### Ferroptosis modulation

#### Sperm motility

**Figure 1:**
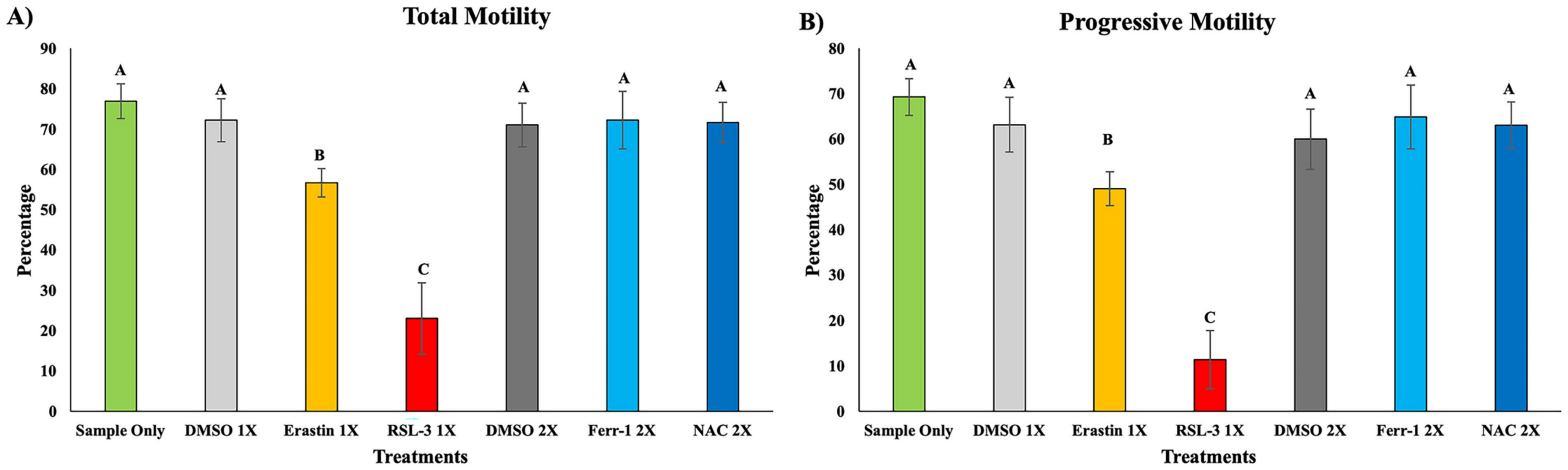
Sperm motility on day 3 of treatment. Sperm motility was evaluated using computer-assisted sperm analysis (CASA) on day 3 of treatment **(A,B)**. **(A)** Total motility. **(B)** Progressive motility. Data are presented as mean ± SEM (*n* = 5 boars). Different superscript letters indicate significant differences among treatment groups (*p* < 0.05).

Total motility (TM) and progressive motility (PM) on day 3 differed markedly across treatment groups (Figures 1A,B). Untreated and vehicle controls (Sample Only, DMSO 1X, DMSO 2X) maintained the highest motility values, comparable to ferroptosis inhibitor treatments (Ferrostatin-1 and NAC). The class I inducer Erastin caused moderate but significant reduction in both TM and PM relative to controls (*p* < 0.05). The GPX4 inhibitor RSL-3 (class II inducer) produced the greatest decline in TM and PM, exhibiting significantly lower motility than all other groups (*p* < 0.05).

#### Single-cell biomarkers reflecting overall sperm population health

**Figure 2:**
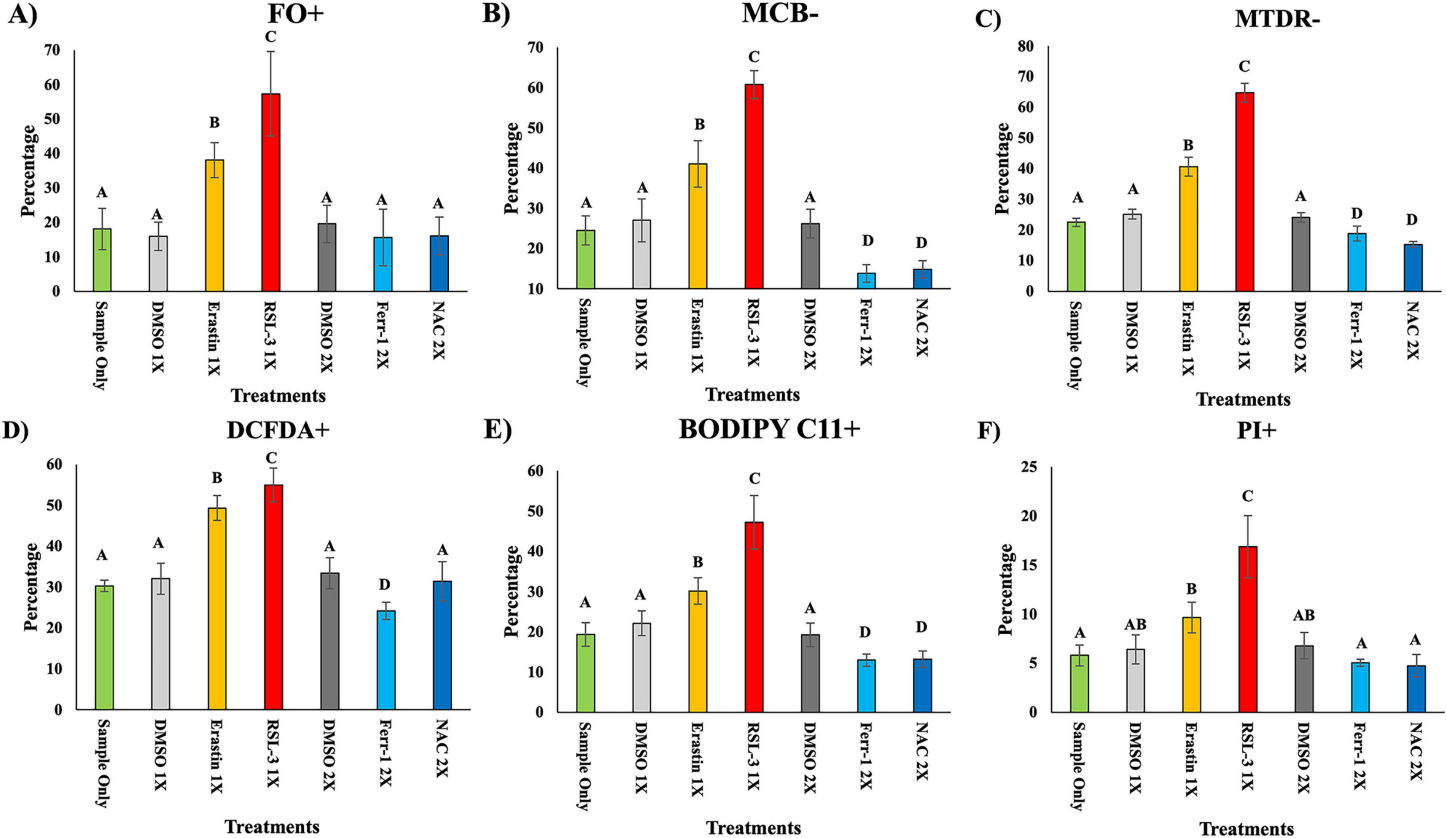
Ferroptosis-associated biomarkers in single sperm cells on day 3. Single sperm cell populations were analyzed for ferroptosis-associated biomarkers on day 3 of treatment **(A–F)**. **(A)** Intracellular ferrous iron (Fe^2+^) accumulation quantified using FerroOrange (FO+). **(B)** Intracellular glutathione depletion assessed using Monochlorobimane (MCB−). **(C)** Mitochondrial membrane potential disruption assessed using MitoTracker Deep Red (MTDR−). **(D)** Total intracellular reactive oxygen species measured using DCFDA (DCFDA+). **(E)** Lipid peroxidation assessed using BODIPY 581/591 C11 (BODIPY C11+). **(F)** Plasma membrane integrity evaluated using Propidium Iodide (PI+). Data are mean ± SEM (*n* = 5 boars). Different superscript letters indicate significant differences among treatment groups (*p* < 0.05). Corresponding statistical values are provided in Supplementary Table S4.

Single-cell analysis of the general sperm population showed clear treatment-dependent differences across biomarker indicators on day 3 (Figures 2A–F). RSL-3 produced the most pronounced disruptions, with significantly higher proportions of FO+ (labile Fe^2+^-accumulating), MCB− (glutathione-depleted), MTDR− (mitochondrial-potential-disrupted), DCFDA+ (ROS-elevated), BODIPY C11+ (lipid-oxidized), and PI+ (membrane-compromised) sperm compared with all other groups (*p* < 0.05). Erastin also increased FO+, MCB−, MTDR−, DCFDA+, and BODIPY C11+ populations relative to untreated and vehicle controls (*p* < 0.05), though the magnitude remained lower than RSL-3. In contrast, Ferrostatin-1 and NAC reduced several adverse biomarker populations, including MCB−, BODIPY C11+, and MTDR−, to levels below untreated controls (*p* < 0.05). These responses indicate strong protective effects of the inhibitors against ferroptotic stress. Complete biomarker expression data for all treatment groups are presented in Supplementary Table S4.

#### Ferroptosis signatures in ferrous iron-accumulating (FO+) sperm populations

The FO+ population represents spermatozoa with elevated intracellular labile Fe^2+^ detected by FerroOrange, a signature consistent with cells entering a ferroptosis-prone state. Excess redox-active iron accelerates ROS generation and lipid peroxidation, making this subset particularly susceptible to ferroptotic progression.

**Figure 3:**
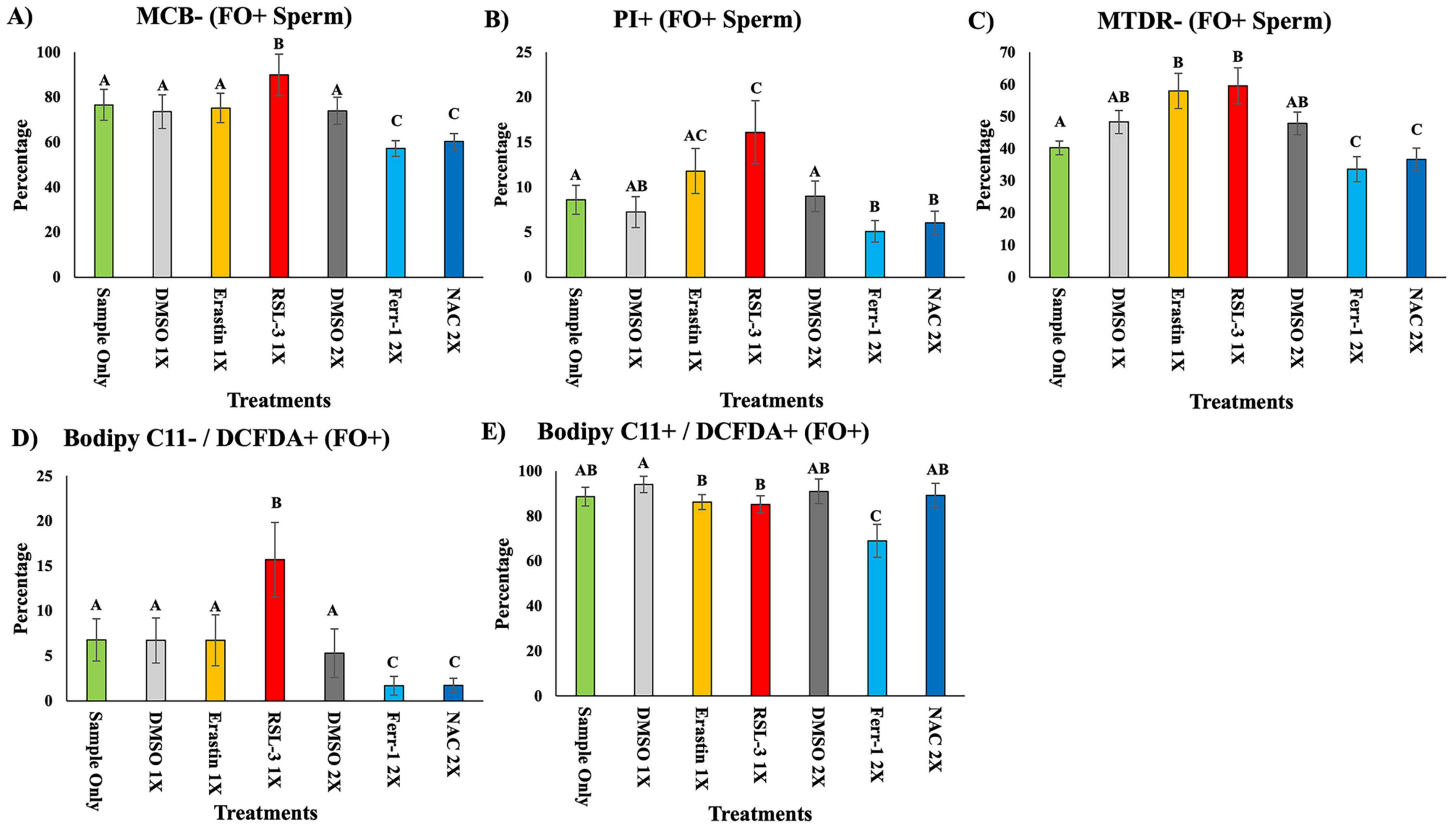
Biomarker profiles in FO+ sperm cells on day 3 of treatment. Sperm populations positive for FerroOrange (FO+; intracellular Fe^2+^) were further analyzed on day 3 of treatment **(A–E)**. **(A)** Glutathione depletion assessed using Monochlorobimane (MCB−). **(B)** Plasma membrane damage assessed using Propidium Iodide (PI+). **(C)** Mitochondrial membrane depolarization assessed using MitoTracker Deep Red (MTDR−). **(D)** BODIPY C11-/DCFDA+ cells representing early oxidative stress progression. **(E)** BODIPY C11+/DCFDA+ cells representing advanced lipid peroxidation consistent with terminal ferroptosis. Data are mean ± SEM (*n* = 5 boars). Different superscript letters indicate significant differences among treatment groups (*p* < 0.05). Corresponding statistical values are provided in Supplementary Table S5.

Within the FO+ population, strong treatment-dependent differences were observed across all biomarkers on day 3 (Figures 3A–E). RSL-3 produced the most severe disruption, yielding the highest proportions of glutathione-depleted (MCB−), plasma membrane-compromised (PI+), and mitochondria-depolarized (MTDR−) FO+ spermatozoa (*p* < 0.05). RSL-3 also drove increases in oxidative signatures, particularly early lipid peroxidation (BODIPY C11−/DCFDA+), indicating pronounced oxidative injury within iron-laden cells (*p* < 0.05).

Erastin produced similar oxidative patterns as untreated controls for several biomarkers but with higher MTDR− levels (*p* < 0.05). In contrast, Ferrostatin-1 and NAC markedly reduced nearly all stressed FO+ subpopulations, including MCB−, BODIPY C11-/DCFDA+, BODIPY C11+/DCFDA+, MTDR−, and PI+ sperm (*p* < 0.05), often falling below untreated baseline levels. These responses indicate strong protection against oxidative and structural injury within the iron-accumulating, ferroptosis-vulnerable subset. Detailed biomarker data for the FO+ population are provided in Supplementary Table S5.

### Untreated semen during liquid storage

#### Motility decline

**Figure 4:**
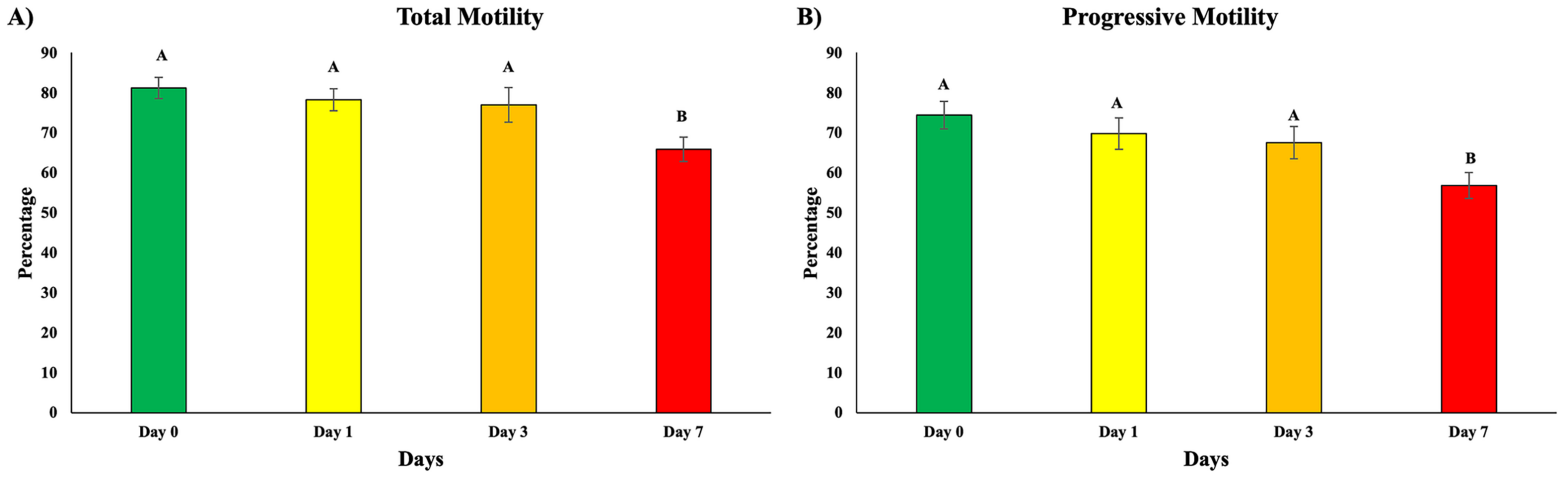
Sperm motility during storage in untreated samples. Sperm motility was evaluated using CASA in untreated semen samples on days 0, 1, 3, and 7 of storage **(A,B)**. **(A)** Total motility. **(B)** Progressive motility. Data are mean ± SEM (*n* = 5 boars). Different superscript letters indicate significant differences among storage days (*p* < 0.05).

To establish the baseline pattern of sperm performance during standard liquid storage and provide context for downstream ferroptosis biomarker analysis, motility was assessed in untreated semen at days 0, 1, 3, and 7 (Figures 4A,B). Total motility and progressive motility were highest on day 0 and remained stable through day 3. By day 7, both TM and PM showed significant decline (*p* < 0.05), confirming the expected late-storage loss of sperm function under routine preservation conditions.

#### Biomarker progression across storage days

**Figure 5:**
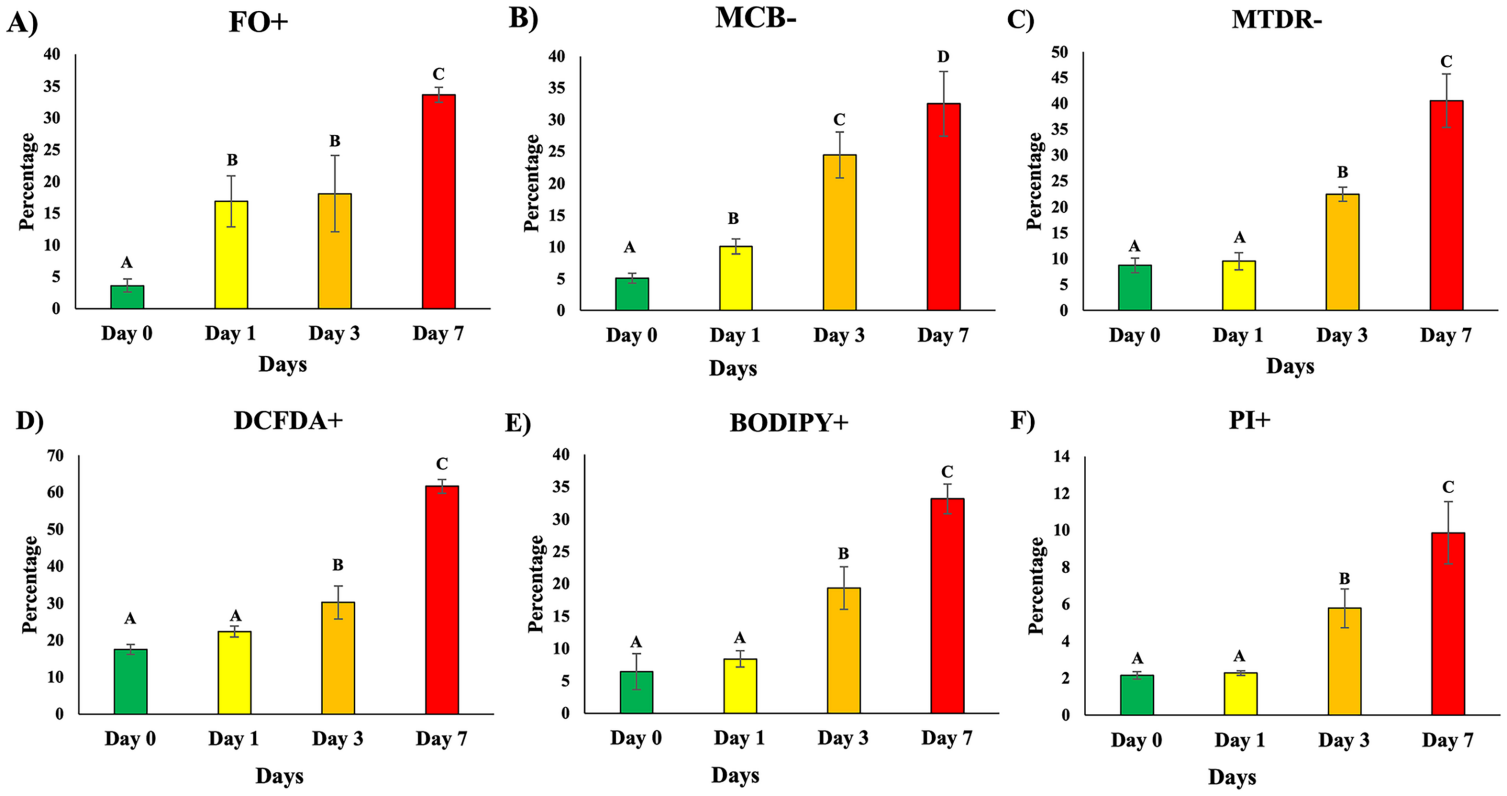
Ferroptosis-associated biomarkers in untreated samples during storage. Single sperm cells from untreated samples were analyzed for ferroptosis-associated biomarkers on days 0, 3, and 7 of storage **(A–F)**. **(A)** FerroOrange (FO^+^; Fe^2+^ accumulation). **(B)** Monochlorobimane (MCB−; glutathione depletion). **(C)** DCFDA (DCFDA+; total ROS). **(D)** BODIPY 581/591 C11 (BODIPY C11+; lipid peroxidation). **(E)** MitoTracker Deep Red (MTDR−; mitochondrial depolarization). **(F)** Propidium Iodide (PI^+^; membrane damage). Data are mean ± SEM (*n* = 5 boars). Different superscript letters indicate significant differences among storage days (*p* < 0.05). Corresponding statistical values are provided in Supplementary Table S6.

Single-cell biomarker analysis of untreated semen revealed clear, time-dependent deterioration in sperm cell health over the 7-day storage period (Figures 5A–F). All biomarkers showed a similar directional pattern: lowest levels on day 0, modest elevations by day 1, and more pronounced shifts by day 3, with each indicator reaching its highest value by day 7 (*p* < 0.05).

Intracellular ferrous iron accumulation (FO+) increased steadily, indicating rising levels of labile Fe^2+^ within the sperm population (*p* < 0.05). Glutathione-depleted cells (MCB−) also expanded over time, reflecting progressive loss of antioxidant capacity (*p* < 0.05). Total reactive oxygen species (DCFDA+) and lipid peroxidation (BODIPY C11+) increased in parallel, suggesting that oxidative pressure intensifies as storage time increases (*p* < 0.05). Mitochondrial membrane depolarization (MTDR−) increased between days 1 and 3 and peaked at day 7, consistent with growing mitochondrial dysfunction (*p* < 0.05). Plasma membrane-compromised sperm (PI+) remained low through day 1, rose by day 3, and showed the largest increase on day 7 (*p* < 0.05). Complete longitudinal biomarker data for untreated controls are presented in Supplementary Table S6.

#### Storage-time progression of ferroptosis indicators in ferrous iron-accumulating sperm (FO+)

**Figure 6:**
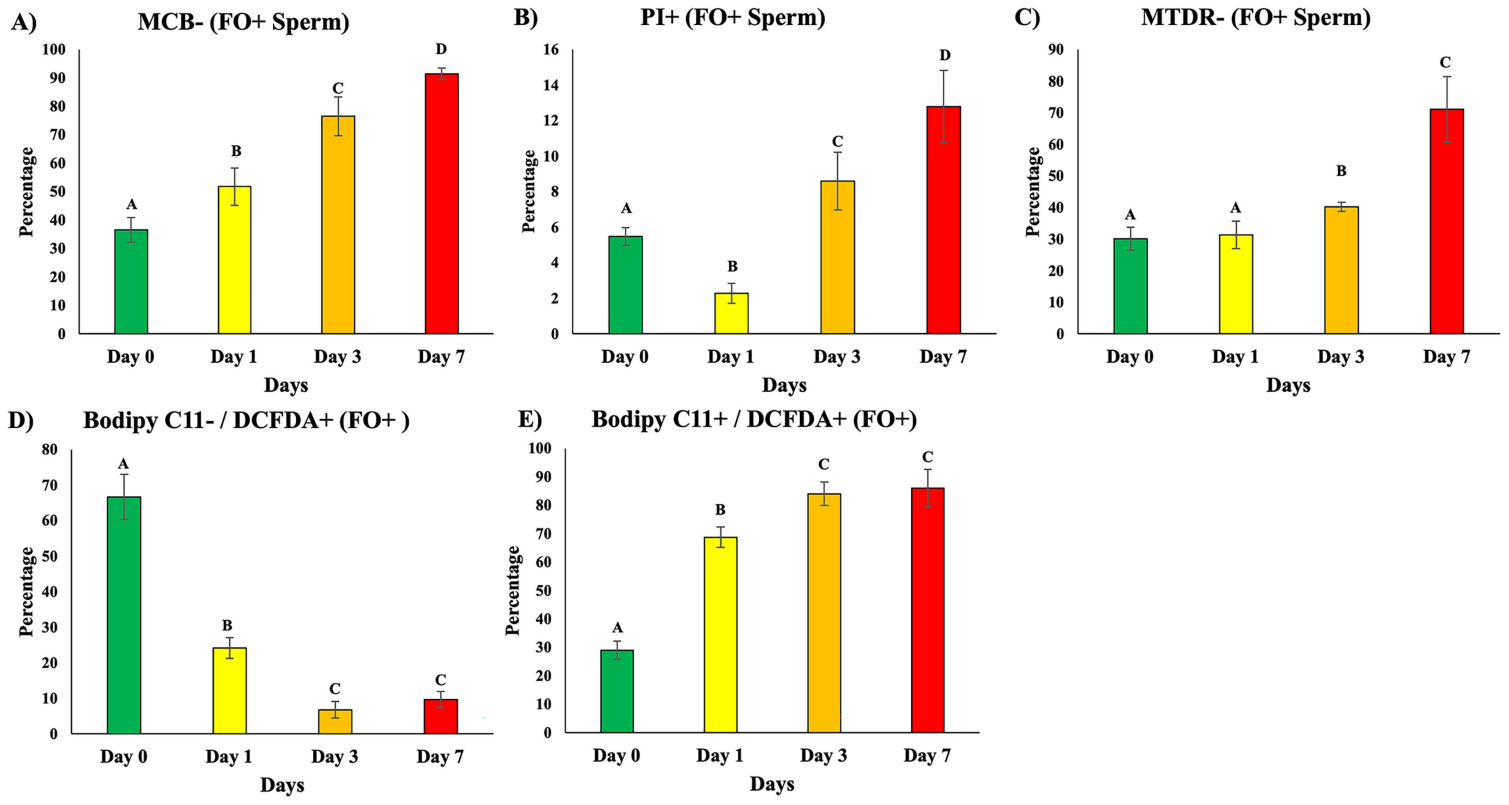
Biomarker profiles in FO+ sperm cells during storage. FO+ sperm populations from untreated samples were analyzed across storage timepoints **(A–E)**. **(A)** Monochlorobimane (MCB−; glutathione depletion). **(B)** Propidium iodide (PI+; membrane damage). **(C)** BODIPY C11−/DCFDA+ cells representing early oxidative stress progression. **(D)** BODIPY C11+/DCFDA+ cells representing advanced lipid peroxidation. **(E)** MitoTracker Deep Red (MTDR−; mitochondrial depolarization). Data are mean ± SEM (*n* = 5 boars). Different superscript letters denote significant differences among storage days (*p* < 0.05). Corresponding statistical values are provided in Supplementary Table S7.

Biomarker analysis within the FO+ sperm subpopulation revealed distinct progression patterns across the 7-day storage period (Figures 6A–E). Glutathione-deficient FO+ sperm (MCB−) were least abundant on day 0 and increased steadily through days 1 and 3, reaching highest levels on day 7 (*p* < 0.05). Plasma membrane-compromised FO+ sperm (PI+) followed a similar pattern, remaining low through day 1 and rising significantly by day 7 (*p* < 0.05). FO+ sperm with disrupted mitochondrial membrane potential (MTDR−) remained low on days 0 and 1 but increased markedly at day 3 and reached highest levels by day 7 (*p* < 0.05). Early-stage oxidative stress (BODIPY C11-/DCFDA+) was most prevalent on day 0 and declined progressively, showing lowest levels on days 3 and 7 (*p* < 0.05). In contrast, FO+ sperm exhibiting concurrent ROS accumulation and lipid peroxidation (BODIPY C11+/DCFDA+) increased over time, with minimal levels on day 0 and highest values on days 3 and 7 (*p* < 0.05). Longitudinal FO+ population biomarker data are provided in Supplementary Table S7.

## Discussion

The current results demonstrate that ferroptosis can be experimentally induced and partially inhibited in boar spermatozoa during liquid semen storage. The observed biochemical signatures (ferrous iron accumulation, glutathione depletion, oxidative damage, and compromised mitochondrial function) collectively establish ferroptosis as a mechanistically relevant pathway contributing to sperm functional decline (2, 5). Significant variations in sperm motility were observed across treatments, with RSL-3 inducing rapid motility decline by day 3, likely through direct GPX4 inhibition triggering early oxidative stress and membrane damage. The distinct mechanisms of these inducers align with established ferroptosis pathway models, where GPX4 inhibition directly allows lipid hydroperoxide accumulation, while system Xc^−^ blockade depletes the GSH substrate needed for GPX4 enzymatic function (32). Erastin, which blocks the cystine/glutamate antiporter system Xc^−^ leading to GSH reduction and subsequent oxidative damage, showed delayed motility decline reaching maximum effect by day 7, consistent with a slower mode of ferroptosis induction. NAC provided greater motility preservation than Ferrostatin-1, consistent with its ability to bolster intracellular redox capacity through glutathione support, while Ferrostatin-1’s partial protection suggests that lipid ROS scavenging alone is insufficient to maintain sperm function under storage conditions.

The distinct effects of RSL-3 and Erastin on sperm ferroptosis reflect their different mechanisms of action. RSL-3 acts by directly inhibiting GPX4, triggering rapid lipid peroxidation and manifest ferroptotic damage by day 3. Erastin acts upstream by blocking the systemic Xc^−^ transporter, inhibiting cystine uptake and resulting in gradual GSH depletion, which requires more time for ferroptosis manifestation. The colocalization analyses of biomarkers within FO+ populations further resolved ferroptosis-associated stages based on oxidative stress, lipid peroxidation, GSH depletion, mitochondrial membrane potential, and membrane integrity. RSL-3-treated groups exhibited the highest glutathione depletion, oxidative stress, and plasma membrane damage in iron-accumulating sperm, reflecting cells in an early redox-deficient state prior to full lipid peroxidation. These populations were significantly lower in ferroptosis inhibitor-treated groups, particularly Ferrostatin-1, suggesting protective effects. The MCB−/PI+ population, representing GSH-depleted sperm with damaged plasma membranes, was significantly higher in RSL-3-treated groups on day 3 and remained elevated through day 7, particularly in Erastin-treated samples, while inhibitor groups showed lower populations than controls, suggesting protective effects.

The weaker overall ferroptotic response to Erastin compared to RSL-3, beyond its delayed kinetics, likely reflects multiple converging factors. Erastin is metabolically labile with low water solubility (33), which may limit its sustained bioactivity in semen extender medium over the multi-day storage period used in this study. Moreover, GSH synthesis in spermatozoa may not be entirely dependent on system Xc^−^-mediated cystine import. In stallion spermatozoa, the SLC7A11 antiporter mediates cystine uptake for GSH synthesis and is critical for mitochondrial function, yet inhibition of SLC7A11 with sulfasalazine, while causing a dramatic drop in intracellular GSH, did not completely eliminate it (34), suggesting that spermatozoa may acquire cysteine through alternative routes. Cystine supplementation increased sperm GSH content by approximately 50%, with sulfasalazine blocking this increase but not reducing GSH below baseline levels (35). In addition, residual cysteine or cystine present in seminal fluids at the time of semen collection may transiently contribute to intracellular thiol pools following extension, although this source is likely diluted and depleted during prolonged storage. In somatic cells, the transsulfuration pathway synthesizes cysteine *de novo* from methionine via cystathionine β-synthase and cystathionine γ-lyase, and activation of this pathway confers resistance to system Xc^−^ inhibition (36). If boar spermatozoa possess even partial transsulfuration activity or alternative cysteine transporters, Erastin-mediated system Xc^−^ blockade would result in incomplete GSH depletion, leaving residual GPX4 activity to buffer against lipid peroxidation. In contrast, RSL-3 bypasses the entire GSH synthesis axis by directly inactivating GPX4, producing immediate and complete loss of the primary lipid hydroperoxide defense regardless of cysteine source. Whether boar spermatozoa express functional transsulfuration enzymes and the extent to which alternative cysteine acquisition pathways modulate ferroptosis susceptibility in stored semen warrant further investigation.

Species-specific ferroptosis susceptibility likely reflects differences in sperm membrane composition, antioxidant capacity, and mitochondrial function. Boar sperm, like mammalian sperm generally, possess polyunsaturated fatty acid-rich plasma membranes that are inherently susceptible to iron-catalyzed lipid peroxidation, but the relative sensitivity compared to human, bull, or stallion sperm may vary based on specific lipid profiles and endogenous antioxidant systems. Understanding these species differences is important for developing species-specific semen preservation strategies. The dual role of reactive oxygen species in sperm function, necessary at physiological levels for capacitation yet damaging at pathological concentrations, further complicates ferroptosis-targeted interventions, as complete suppression of oxidative signaling may impair normal sperm function (13), as the optimal ferroptosis-inhibitor concentration and extender formulation may differ significantly across species.

These findings have significant implications for the optimization of semen extender formulations. Current extenders typically include antioxidant cocktails (vitamins E and C, catalase, superoxide dismutase), but ferroptosis-specific strategies have not been systematically incorporated. Future extender development might include: iron chelators to prevent labile iron accumulation; ferroptosis inhibitors such as ferrostatin-1 or liproxstatin-1 to block lipid peroxidation; glutathione (GSH) or GSH precursors (like N-acetylcysteine) to restore antioxidant capacity; and synergistic combinations targeting multiple steps in the ferroptosis cascade. The dose-dependent relationships, timing of inhibitor exposure, and potential interactions with existing extender components require systematic evaluation to optimize efficacy while maintaining regulatory compatibility and cost-effectiveness. Recent advances in boar semen preservation have explored diverse antioxidant approaches including nanotechnology-based delivery systems and Nrf2 pathway activators (17, 37). The development of antibiotic-free extenders with organic bactericidal compounds represents an additional dimension of extender optimization that must be considered alongside ferroptosis-targeted strategies (38).

Ferroptosis likely represents one of multiple regulated cell death pathways (4) operating in sperm, and crosstalk between ferroptosis, apoptosis, and necroptosis pathways likely occurs during liquid storage. The present study focused specifically on ferroptosis markers, but future investigations should evaluate whether ferroptosis inhibition reduces apoptotic and necroptotic markers, and whether apoptosis or necroptosis inhibition affects ferroptosis progression. Such mechanistic understanding would clarify whether combined pathway inhibition could provide superior sperm protection compared to single-pathway targeting. A recent comprehensive review of regulated cell death in sperm cryoinjury emphasizes the interconnected nature of ferroptosis, apoptosis, and necroptosis pathways, with shared upstream triggers including mitochondrial dysfunction and calcium dysregulation (39). The interplay between ferroptosis and other RCD pathways including cuproptosis and pyroptosis has been increasingly recognized in male infertility contexts (40). Additionally, the relative contribution of ferroptosis versus oxidative damage from other mechanisms to the overall loss of sperm viability during storage requires quantitative determination.

The economic implications of ferroptosis-targeted preservation strategies could be substantial for the swine industry. Over 95% of pig production in developed countries relies on artificial insemination using liquid-stored semen, with typical storage at 15–17 °C for 3–7 days. Each 5% improvement in post-storage sperm viability or fertility translates to significant potential economic returns with less open sows/gilts or increased litter sizes. The swine industry has invested heavily in genetic improvement through artificial insemination, and preservation of sperm quality directly impacts reproductive efficiency and profitability. Enhanced semen storage techniques could improve the efficiency of elite boar utilization in breeding programs by enabling fewer sperm per dose.

Having established that ferroptosis can be experimentally induced in boar sperm using RSL-3 and Erastin, the critical finding was that a similar process emerges naturally during liquid storage. In untreated control groups, FO+, MCB−, DCFDA+, BODIPY C11+, and MTDR− populations increased steadily from day 0 to day 7, indicating that sperm progressively accumulate ferroptosis-associated stress even without chemical induction. This coordinated shift across multiple biomarkers (labile Fe^2+^ buildup, glutathione depletion, mitochondrial dysfunction, membrane permeability loss, and lipid peroxidation) suggests a ferroptosis-like process unfolds during standard storage conditions. The lipid-rich sperm membranes, low cytoplasmic antioxidant defenses, and dependence on mitochondrial energy production create an ideal environment for ferroptotic activation. During liquid storage, intracellular redox imbalance appears to destabilize iron homeostasis within sperm. Li et al. (16) demonstrated that ferroptosis inhibition with liproxstatin-1 significantly improved boar sperm quality during liquid storage at 17 °C by reducing oxidative stress and ferroptosis markers, consistent with the ferroptotic progression observed in the present study, where labile Fe^2+^ accumulation reflects intracellular redox imbalance rather than an iron-rich seminal environment. Recent studies using liproxstatin-1 have demonstrated that ferroptosis inhibition significantly improves sperm quality during liquid storage at 17 °C, supporting the mechanistic relevance of ferroptosis to storage-related sperm damage.

Ferroptosis has been reported to contribute to sperm damage during both cryopreservation and liquid storage in multiple species. In goats, ferroptosis was identified as the predominant regulated cell death pathway during semen cryopreservation based on ferroptosis markers and demonstrated improvement in post-thaw sperm function after ferroptosis inhibitor treatment (26). Similarly, in rams, inhibiting ferroptosis during semen freezing with ferrostatin-1 improved post-thaw sperm motility, mitochondrial function, acrosome integrity, and plasma membrane integrity through partial GPX4 restoration (27). In conjunction with the current boar data, these studies across multiple species suggest that ferroptosis plays a significant and conserved role in causing sperm damage during both liquid storage and cryopreservation. Oxidative stress, membrane disruption, and mitochondrial dysfunction have been extensively reviewed as primary mechanisms of cryodamage in boar sperm (41). Notably, these cryodamage mechanisms overlap with hallmarks of ferroptosis, providing important context for distinguishing ferroptosis-specific injury from general preservation damage.

In the present study, NAC and Ferrostatin-1 each provided measurable but moderate protection against ferroptosis-associated stress, including reductions in FO+, MCB−, BODIPY C11+, MTDR−, and PI+ populations and partial preservation of motility. These findings suggest that pharmacological modulation of ferroptosis is feasible in ejaculated sperm and that NAC and Ferrostatin-1 warrant further evaluation as semen extender additives for improved boar semen preservation. However, further exploration of dose-dependent effects, timing of inhibitor exposure relative to collection and processing, targeted molecular assays (e.g., GPX4 expression or lipid peroxide quantification), potential off-target effects on sperm function unrelated to ferroptosis, and downstream impacts on *in vivo* fertility and embryo development are essential before translational applications. Computer-aided semen analysis parameters including motility have been shown to correlate with field fertility outcomes in pigs (42), providing a framework for evaluating whether ferroptosis inhibitor supplementation translates to improved reproductive performance in artificial insemination systems. Additionally, the stability of these compounds in semen extender during storage and interactions with other extender components must be addressed.

## Conclusion

This study demonstrates that ferroptosis can be experimentally induced in boar spermatozoa, with RSL-3 triggering rapid and pronounced ferroptotic damage by day 3 and Erastin producing a slower, delayed response reaching maximum by day 7, while NAC and Ferrostatin-1 provided measurable but partial protection, confirming that ferroptosis is both inducible and pharmacologically modifiable in boar sperm. In parallel, untreated semen exhibited a coordinated, time-dependent rise in labile Fe^2+^ accumulation, glutathione depletion, oxidative stress, mitochondrial depolarization, and membrane disruption from day 0 through day 7, indicating that a ferroptosis-like process emerges naturally during liquid storage. Together, these inducible and spontaneous patterns establish ferroptosis as a mechanistically relevant contributor to sperm deterioration during liquid semen storage and provide a rational foundation for developing ferroptosis-targeted preservation strategies to improve sperm quality and fertility outcomes in swine artificial insemination systems. Future research should focus on optimizing ferroptosis inhibitor doses and delivery in semen extenders, evaluating the contribution of ferroptosis relative to other RCD pathways, assessing the impact of ferroptosis inhibition on *in vivo* fertility and embryonic development, and determining the cost–benefit analysis for commercial implementation.

## Data Availability

The raw data supporting the conclusions of this article will be made available by the authors, without undue reservation.
