# Supplementary material for: Ferroptosis contributes to boar sperm deterioration during liquid semen storage
Source: Front Vet Sci. 2026 Apr 22;13:1816003. doi: 10.3389/fvets.2026.1816003 (PMC13143747; doi:10.3389/fvets.2026.1816003)
Supplement: Supplementary file 1 [file Data_sheet_1.docx]

## Supplemental Data

### Supplementary Methods

The fluorophores used in the study were reconstituted using the following information:

#### FerroOrange (FO) Fluorescent Probe

FerroOrange (FO) fluorescent probe from Dojindo™ (F374) was reconstituted using DMSO to make a 1 mM stock solution. For this purpose, 35 µL of DMSO was added to 24 µg of FO. The fluorophore was vortexed to mix thoroughly. The reconstituted fluorophore was divided into 10 µL aliquots and stored at 5 ℃.

#### Propidium Iodide (PI) Fluorescent Probe

Propidium Iodide (PI) Fluorescent Probe from Thermo Scientific™ (25535-16-4) was reconstituted using ddH_2_O to make a 1 mg/mL stock solution. For this purpose, 1 mL of ddH_2_O was added to 1 mg of PI. The fluorophore was vortexed to mix thoroughly, divided into 10 µL aliquots, and stored at 5℃.

#### Monochlorobimane (MCB) Fluorescent Probe

Monochlorobimane (MCB) fluorescent probe from Sigma-Aldrich (BCCB4880) using DMSO to make a 100 mM stock solution. For this purpose, 220.6 µL of DMSO was added to 5 mg of MCB. The fluorophore was vortexed to mix thoroughly; the stock solution was divided into 10 µL aliquots and stored at -20℃. A working secondary dilution was made before time of use, with the secondary stock being 1-part MCB with 99 parts ddH_2_O (1 uL MCB + 99 uL ddH_2_O). The fluorophore was vortexed to mix thoroughly, divided into 10 µL aliquots, and stored at -20℃.

#### Hoechst 33342 (H33342) Fluorescent Probe

Hoechst 33342 (H33342) fluorescent probe from Calbiochem (382065) was reconstituted using ddH_2_O to make an 18 mM stock solution in 1000 µL of ddH_2_O. The fluorophore was vortexed to mix thoroughly, divided into 50 µL aliquots, and stored at 5 ℃.

#### BODIPY 581/591 C11 Fluorescent Probe

BODIPY 581/591 C11 fluorescent probe from Invitrogen™ (D3861) was reconstituted using DMSO to make a 5 mM stock solution. For this purpose, 396.51 µL of DMSO was added to 1 mg of BODIPY. The fluorophore was vortexed to mix thoroughly, divided into 20 µL aliquots, and stored at -20℃. A 50µM working secondary dilution was made before time of use, with the secondary stock being 2 parts BODIPY and 198 parts ddH_2_O (0.2 µL BODIPY + 19.8 µL ddH_2_O).

#### BODIPY 665/676 Fluorescent Probe

BODIPY 665/676 fluorescent probe from Invitrogen™ (2897770) was reconstituted using DMSO to make a 25 mM stock solution. For this purpose, 446.11 µL of DMSO was added to 5 mg of BODIPY. The fluorophore was vortexed to mix thoroughly, divided into 10 µL aliquots, and stored at -20℃. A working secondary dilution was made before time of use, with the secondary stock being 1 part BODIPY and 199 parts ddH_2_O (0.25 µL BODIPY + 49.75 µL ddH_2_O).

#### MitoTracker® Deep Red (MTDR) Fluorescent Probe

MitoTracker® Deep Red (MTDR) fluorescent probe from Invitrogen (M22426) was reconstituted using DMSO to make a 1 mM stock solution.  For this purpose, 92 µL of DMSO was added to 50 µg MTDR. The fluorophore was vortexed to mix thoroughly, divided into 10 µL aliquots, and stored at -20℃.

#### 6-carboxy-2',7'-dichlorodihydrofluorescein diacetate (Carboxy DCFDA) Fluorescent Probe

6-carboxy-2',7'-dichlorodihydrofluorescein diacetate (Carboxy DCFDA) Fluorescent Probe from Invitrogen was reconstituted using DMSO to make a 200 millimolar (mM) stock solution. For this purpose, 47.25 µL of DMSO was added to 5 milligrams (mg) of carboxy DCFDA. The fluorophore was vortexed to mix thoroughly. The stock solution was divided into 10 µL aliquots and stored at -20℃. A 5 mM secondary dilution was made, with the stock being 5 parts carboxy DCFDA and 195 parts DMSO (1.25 µL carboxy DCFDA + 48.75 µL DMSO). This secondary stock solution was vortexed to mix thoroughly, divided into 10 µL aliquots, and stored at -20℃. A 500µM tertiary working dilution was made before the time of use, with the secondary stock being 10 parts carboxy DCFDA and 90 parts PBS (5 µL carboxy DCFDA + 45 µL PBS). The working solution was vortexed to mix thoroughly, divided into 10 µL aliquots, and stored at 4℃.

#### 4,5-diaminofluorescein diacetate (DAF-2 DA) fluorescent probe

4,5-diaminofluorescein diacetate (DAF-2 DA) fluorescent probe from Chemodex (D0085) was reconstituted using DMSO to make a 5 millimolar (mM) stock solution. For this purpose, 448.1 µL of DMSO was added to 1milligram (mg) of DAF-2 DA. The fluorophore was vortexed to mix thoroughly. The stock solution was divided into 20 µL aliquots and stored at -20℃.

### Supplementary Figures


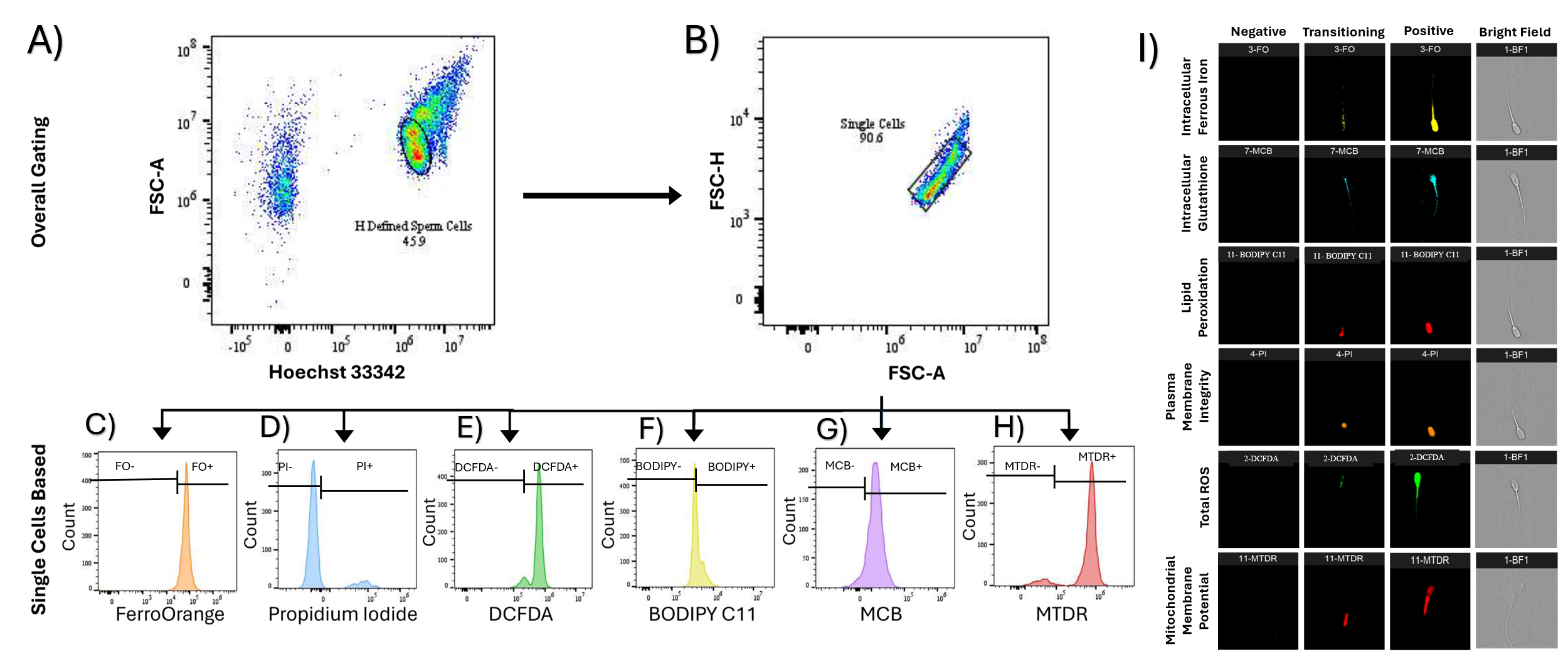
**Supplementary Figure S1. Gating strategy and representative fluorescence profiles for spectral flow cytometry (SFC) and image-based single-cell analysis of boar spermatozoa.** Representative gating strategy for spectral flow cytometry (SFC) analysis in boar sperm populations (A-H). Sequential gating strategy (A-B) in FlowJo (v10) used to identify single sperm populations. Hoechst 33342 fluorescence (DNA content) was used to discriminate sperm cells from debris, followed by doublet exclusion using forward scatter area versus height (FSC-A vs. FSC-H) to obtain single-cell events. Representative histograms (C-H) showing fluorescence intensity distribution for each fluorophore in the single-sperm population: (C) FerroOrange (Fe²⁺ accumulation), (D) Propidium Iodide (PI; plasma membrane integrity), (E) DCFDA (total reactive oxygen species), (F) BODIPY 581/591 C11 (lipid peroxidation), (G) Monochlorobimane (MCB; reduced glutathione), and (H) MitoTracker Deep Red (MTDR; mitochondrial membrane potential). Gates for negative and positive populations were determined using unstained and single-stained controls to ensure accurate fluorescence thresholding across assays. Panel I represents the corresponding image gallery from IBFC depicting representative single-sperm fluorescence patterns for each fluorophore, confirming the cellular localization and emission profile of the respective probes.


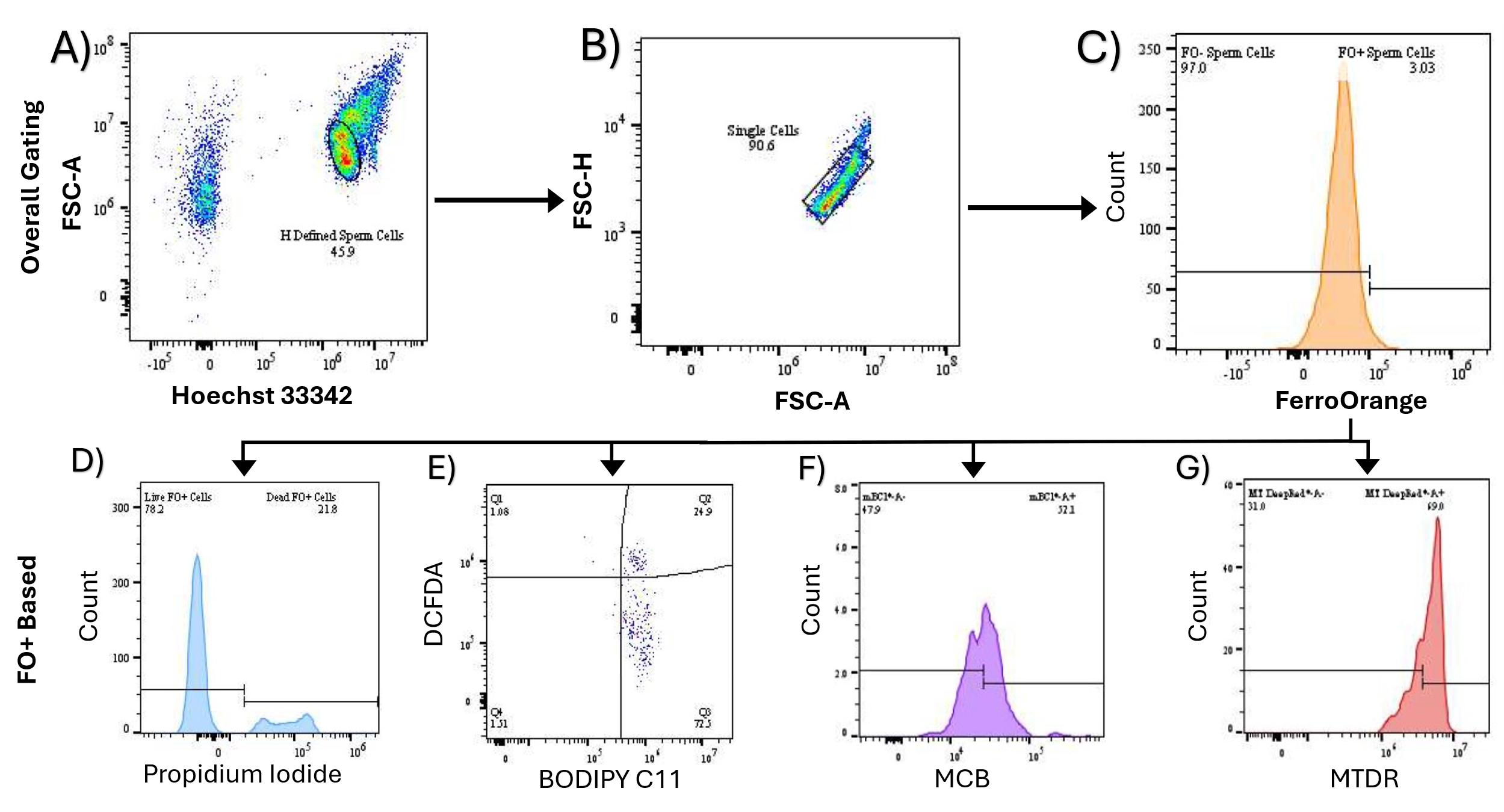


**Supplementary Figure S2.** **Representative gating strategy for spectral flow cytometry (SFC) analysis of FO⁺ sperm cell populations in boar semen.** The top row illustrates the sequential gating hierarchy used to define the single-cell sperm population (A-C). Hoechst 33342 fluorescence (DNA content) was first used to identify sperm cells (panel A), followed by gating for single-cell events based on forward- and side-scatter (FSC-A vs. FSC-H; panel B). The final gate was set on FerroOrange (FO) fluorescence to distinguish FO⁺ (Fe²⁺-accumulating) sperm cells from FO⁻ sperm cells (panel C). The bottom row illustrates the subsequent gating strategy for FO⁺ sperm cells (panels D-G). FO⁺ sperm cells were further evaluated for (D) Propidium Iodide (PI; plasma membrane integrity), (E) DCFDA versus BODIPY 581/591 C11 (total reactive oxygen species and lipid peroxidation), (F) Monochlorobimane (MCB; intracellular glutathione), and (G) MitoTracker Deep Red (MTDR; mitochondrial membrane potential) fluorescence. This multivariate analysis allowed for a comprehensive assessment of ferroptosis-related changes within the FO⁺ sperm cell population.


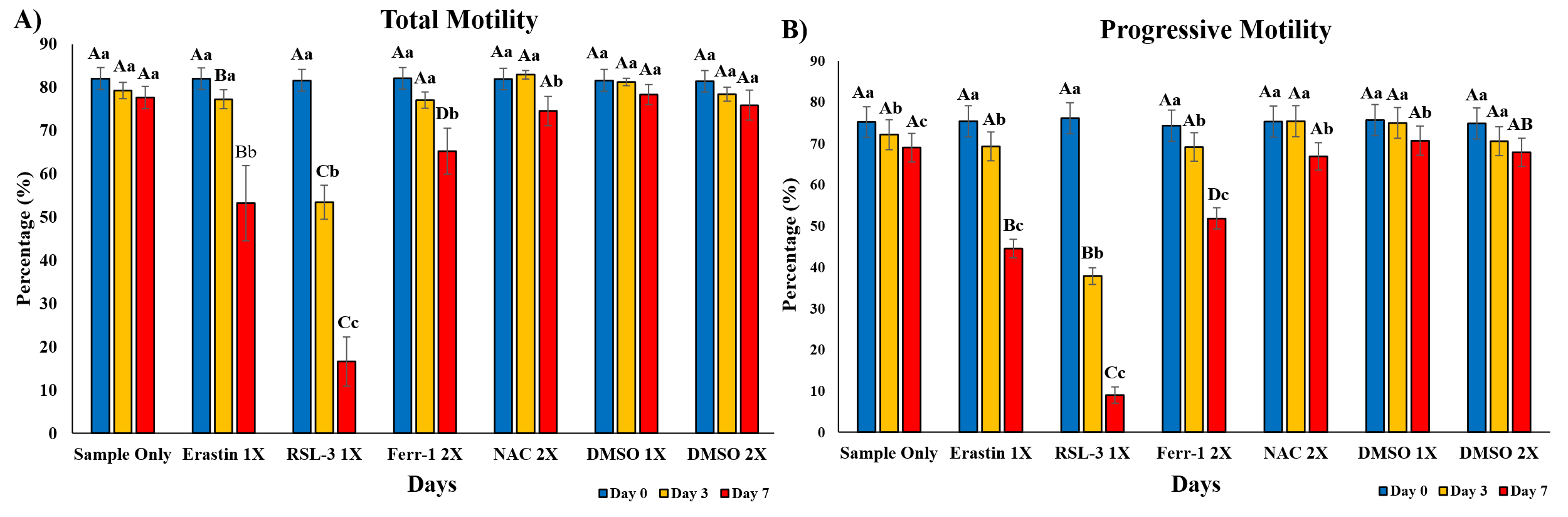
**Supplementary Figure S3. Sperm Motility Results for IBFC Analysis.** Sperm motility was assessed using a Computer-Assisted Semen Analyzer (CASA) for different treatment samples on days 0, 3, and 7 of storage (A-B). (A) represents the total motility in sperm cells, while (B) represents the progressive motility in sperm cells. Bars corresponding to Day 0, Day 3, and Day 7 are represented in blue, yellow, and red color bars, respectively. Data are presented as mean ± SEM; n=6 boars. Uppercase letters indicate differences between different treatment groups, while lowercase letters indicate differences within a treatment group. Different letter superscripts indicate significant differences between the treatment groups (p<0.05).


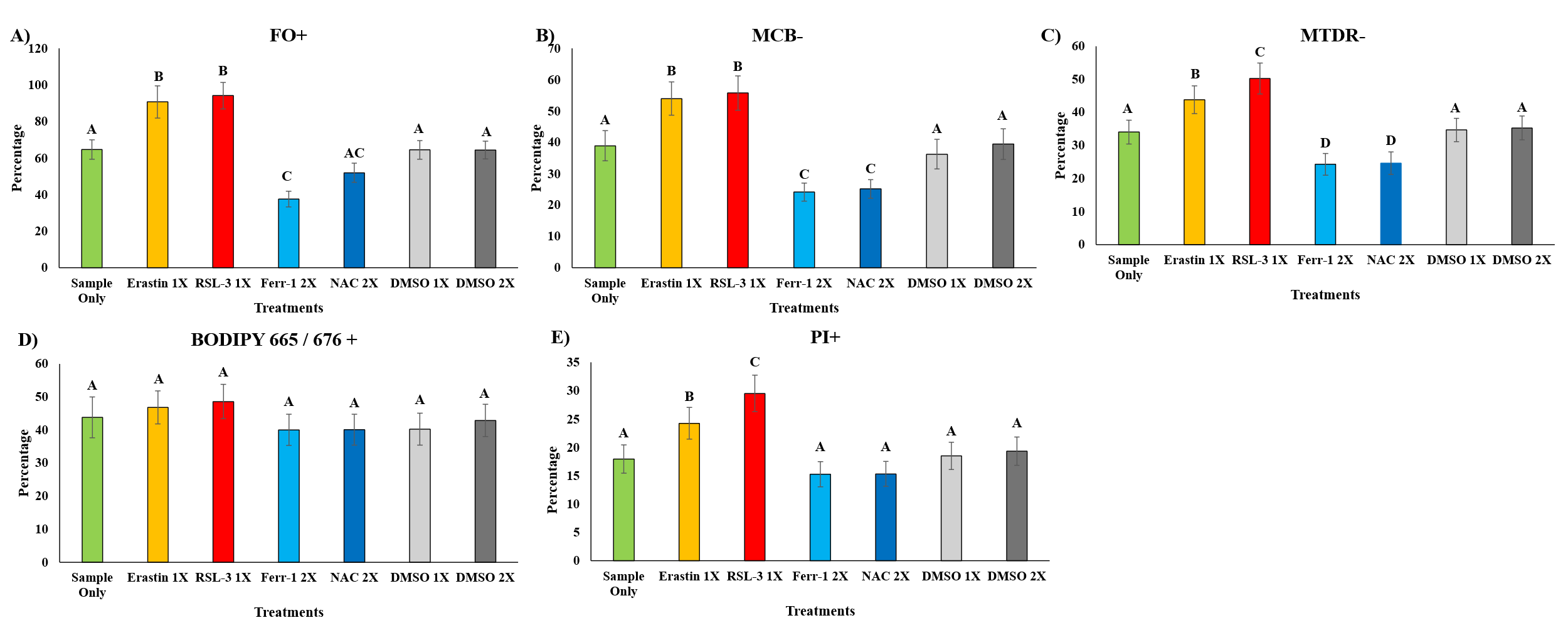
**Supplementary Figure S4. Biomarker Status in Single Cell Population on Day 7 of the treatment.** Single-cell sperm populations were analyzed for different ferroptosis biomarker fluorescence (IBFC) in different treatment groups on day 7 of treatment (A-E). A) represents intracellular ferrous iron accumulation assessed through FerroOrange (FO+). B) represents the intracellular Glutathione (GSH) depletion in sperm cells assessed by Monochlorobimane (MCB-). C) represents disrupted mitochondrial membrane potential assessed by MitoTracker Deep Red (MTDR-). D) represents lipid peroxidation in sperm cells assessed by BODIPY 665/676 (BODIPY 665/676+). Panel E) represents the plasma membrane disruption in sperm cells assessed by Propidium Iodide (PI+). Data are presented as mean ± SEM; n=6 boars. Different letter superscripts indicate significant differences between the treatment groups (p<0.05).


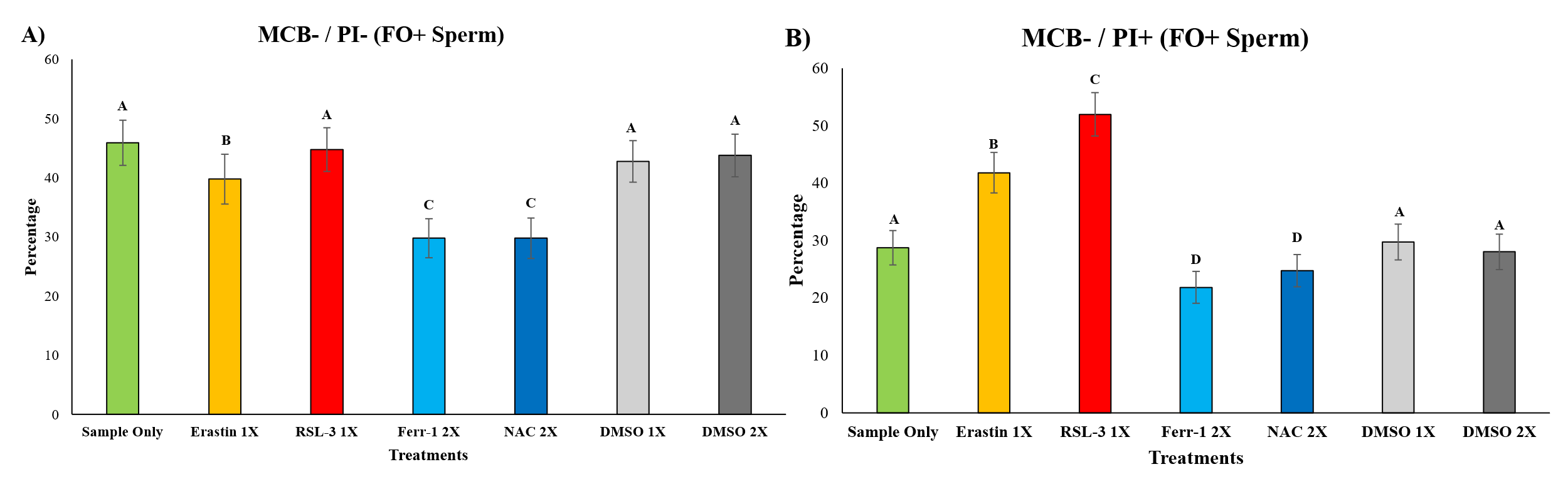
**Supplementary Figure S5. Co-Expression of Monochlorobimane (MCB) and Propidium Iodide (PI) in FO Positive Sperm Cells on Day 7 of Treatment****.** Sperm populations positive for FerroOrange (FO) were analyzed for MCB and PI fluorescence to assess intracellular glutathione (GSH) levels and membrane integrity, respectively (A-B). MCB-/PI- represents early-stage ferroptosis (A) and MCB-/PI+ represents terminal ferroptosis (B). Data are presented as mean ± SEM; n=6 boars, for Day 7of the treatment. Different letter superscripts indicate significant differences between treatments (p<0.05).


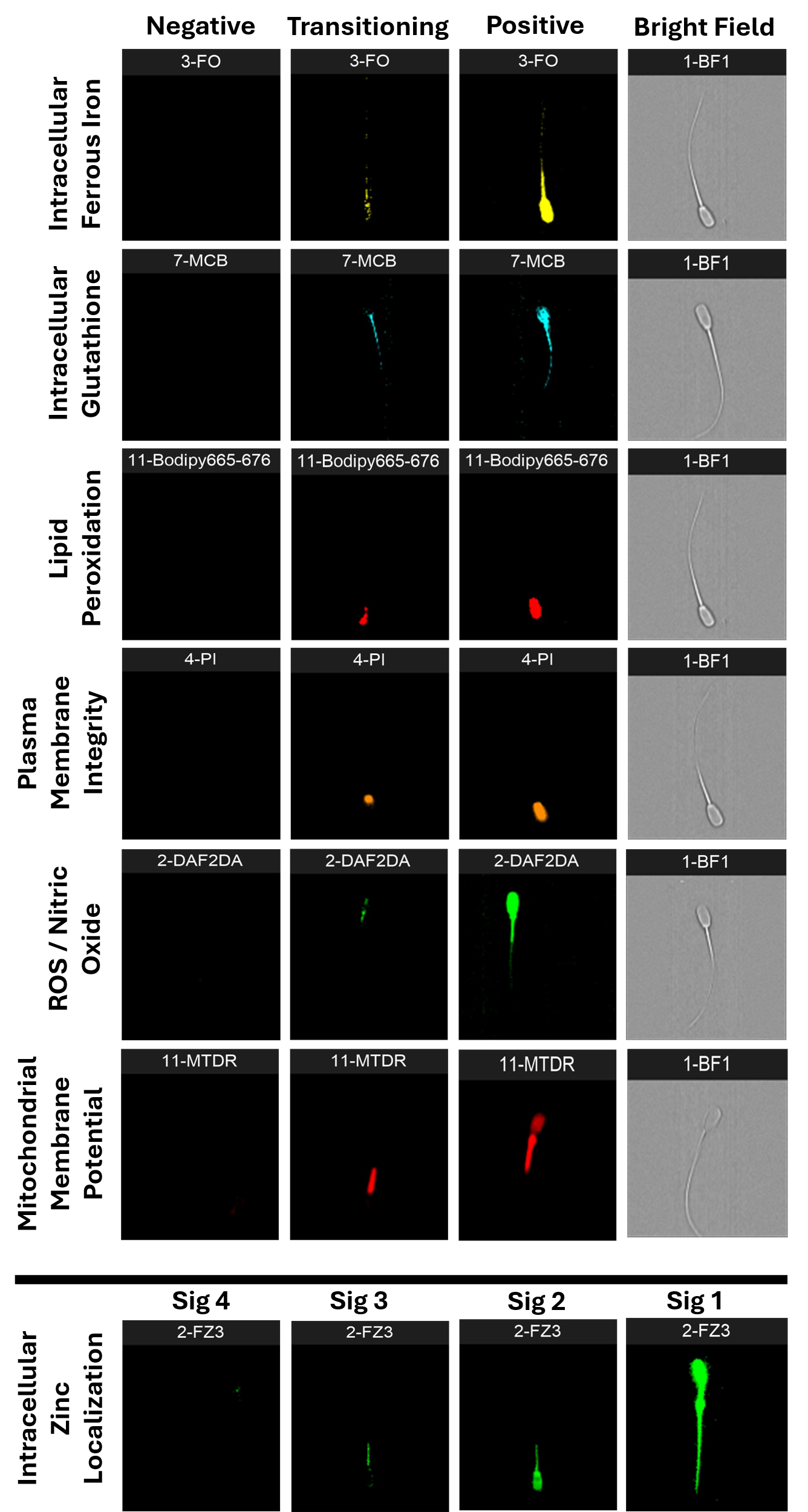


**Supplementary Figure S6. Biomarker Fluorescence Localization Pattern in Boar Sperm Cells.** Representative fluorescence images of boar sperm captured using Image-Based Flow Cytometry (IBFC) on the Amnis ImageStreamX Mk II. Each row illustrates a distinct biomarker associated with ferroptosis or sperm cell health: FerroOrange (FO; ferrous iron accumulation), Monochlorobimane (MCB; intracellular glutathione), BODIPY 665/676 (lipid peroxidation), Propidium Iodide (PI; plasma membrane integrity), DAF-2 DA (nitric oxide), MitoTracker Deep Red (MTDR; mitochondrial membrane potential), and FluoZin-3 (FZ3; intracellular zinc localization). The columns represent negative, transitioning, and positive populations (left to right) based on fluorophore localization in distinct sperm compartments. The final column presents corresponding brightfield (BF) images displaying overall sperm morphology (except for zinc localization). The final row presents different zinc signature patterns based on intracellular zinc localization in sperm cells.


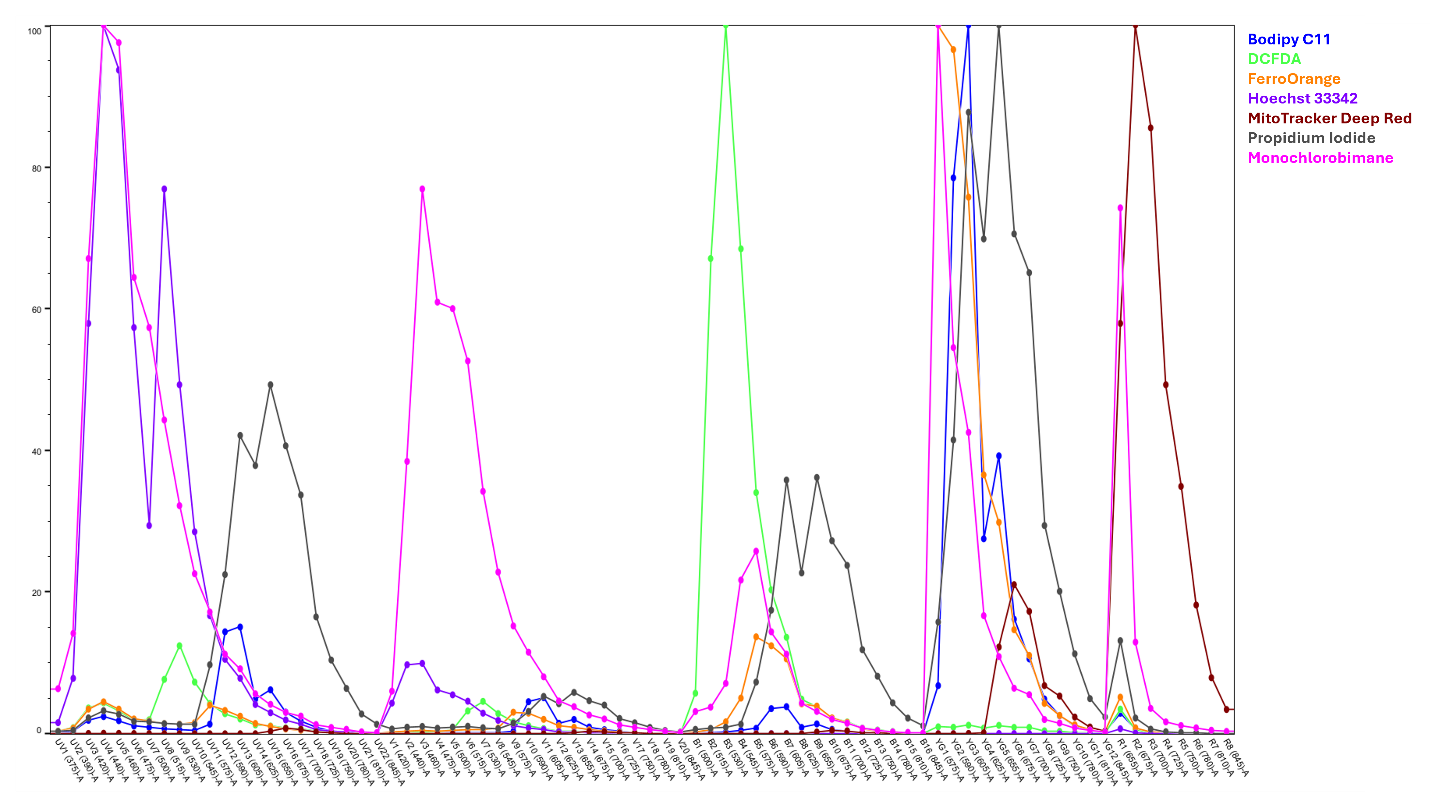


**Supplementary Figure S7. Spectral emission profiles of fluorophores used in the Spectral Flow Cytometry (SFC) panel.** Spectral plot displaying the normalized emission spectra of fluorophores incorporated in the SFC panel for multi-parameter sperm analysis. Each curve represents the emission intensity of an individual fluorophore across the visible spectrum: BODIPY C11, DCFDA, FerroOrange, Hoechst 33342, MitoTracker Deep Red, Propidium Iodide, and Monochlorobimane. The overlapping emission profiles illustrate the importance of spectral unmixing and compensation to accurately resolve fluorophore signals during acquisition.


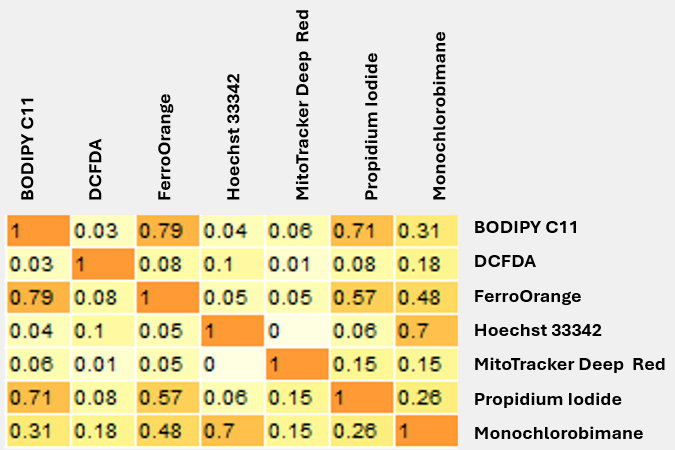


**Supplementary Figure S8. Correlation matrix of fluorophores used in the Spectral Flow Cytometry (SFC) panel.** Correlation matrix illustrating pairwise relationships between the fluorophores in the SFC panel based on their emission spectra. Each cell represents the Pearson correlation coefficient between two fluorophores, with values closer to 1 indicating stronger spectral overlap. Warmer colors (orange/yellow) indicate higher correlation, while cooler colors represent minimal overlap. The panel includes BODIPY C11, DCFDA, FerroOrange, Hoechst 33342, MitoTracker Deep Red, Propidium Iodide (PI), and Monochlorobimane (MCB). This correlation analysis was used to evaluate fluorophore compatibility and optimize spectral unmixing performance.

### Supplementary Tables

**Supplementary Table S1. IBFC Ferroptosis Biomarker Panel 1 and Acquisition Settings.**

| **Fluorophore** | **Concentration** | **Parameter** | **Power (mW)** | **Laser** | **Image Acquisition Channel** |
| --- | --- | --- | --- | --- | --- |
| 4,5-diaminofluorescein diacetate (DAF-2 DA) | 10 µM | ROS production; presence of nitric oxide (NO˙) | 75 | 488 | 2 |
| Ferro Orange (FO) | 2.5 µM | Intracellular Ferrous Iron (Fe^2+^) | 100 | 561 | 3 |
| Propidium Iodide (PI) | 1 µM | Viability | 100 | 561 | 4 |
| Monochlorobimane (MCB) | 1 µM | Intracellular glutathione (GSH) | 75 | 405 | 7 |
| BODIPY 665/676 | 187.5 nM | ROS production; Effect of ROS on lipid peroxidation | 100 | 642 | 11 |

Fluorophores included in Panel 1 are listed with their biological targets, excitation laser power, and corresponding ImageStreamX Mk II acquisition channels used for single-cell imaging.

**Supplementary Table S2. IBFC Ferroptosis Biomarker Panel 2 and Acquisition Settings.**

| **Fluorophore** | **Concentration** | **Parameter** | **Power (mW)** | **Laser** | **Image Acquisition Channel** |
| --- | --- | --- | --- | --- | --- |
| FluoZin ™-3 AM (FZ3) | 1 µM | Intracellular Zinc (Zn^2+^) | 50 | 488 | 2 |
| Ferro Orange (FO) | 2.5 µM | Intracellular Ferrous Iron (Fe^2+^) | 100 | 561 | 3 |
| Propidium Iodide (PI) | 1 µM | Viability | 100 | 561 | 4 |
| Hoechst 33342 (H33342) | 18 µM | Nuclear Events | 10 | 405 | 7 |
| MitoTracker Deep Red (MTDR) | 1 µM | Mitochondrial function; mitochondrial membrane potential | 25 | 642 | 11 |

Fluorophores included in Panel 2 are listed with their biological targets, excitation laser power, and ImageStreamX Mk II acquisition channels used for single-cell imaging.

**Supplementary Table S3. Ferroptosis Biomarker Panel (SFC Acquisition settings).** Fluorophores included in the SFC ferroptosis panel are listed with their biological targets, excitation and emission wavelengths, and the corresponding BD FACSDiscover A8 laser channels used for data acquisition.

| **Fluorophore** | **Parameter** | **Excitation (nm)** | **Emission (nm)** | **Laser** |
| --- | --- | --- | --- | --- |
| Hoechst 33342 | Nuclear staining, sperm identification | 355 | 461 | 355 |
| Carboxy-DCFDA | Total ROS | 495 | 529 | 488 |
| FerroOrange (FO) | Intracellular Ferrous Iron (Fe^2+^) | 543 | 580 | 561 |
| Propidium Iodide (PI) | Viability | 532 | 617 | 561 |
| Monochlorobimane (MCB) | Intracellular GSH | 390 | 478 | 405 |
| BODIPY C11 (581/591) | Lipid peroxidation | 581 | 591 | 561 |
| MitoTracker Deep Red (MTDR) | Mitochondrial membrane potential | 641 | 662 | 638 |

**Supplementary Table S4. Single-Cell Biomarkers Reflecting Overall Sperm Population Health.**

| **Treatment** | **FO+** | **MCB-** | **DCFDA+** | **BODIPY C11+** | **MTDR-** | **PI+** |
| --- | --- | --- | --- | --- | --- | --- |
| Sample Only | 18.072 ± 6.01^A^ | 24.46 ± 3.61^A^ | 30.24 ± 1.4^A^ | 19.35 ± 3.29^A^ | 22.48 ± 1.4^A^ | 5.78 ± 1.05^A^ |
| DMSO 1X | 15.964 ± 4.11^A^ | 27 ± 5.33^A^ | 32.02 ± 3.81^A^ | 22.12 ± 4.31^A^ | 25.16 ± 1.63^A^ | 6.39 ± 1.49^AB^ |
| Erastin 1X | 40.09 ± 5.09^B^ | 41.06 ± 5.79^B^ | 49.32 ± 3.048^B^ | 30.12 ± 4.43^B^ | 40.62 ± 3.11^B^ | 9.632 ± 1.57^B^ |
| RSL-3 1X | 57.3 ± 12.27^C^ | 60.78 ± 3.47^C^ | 54.92 ± 4.14^C^ | 47.24 ± 6.67^C^ | 64.74 ± 3.06^C^ | 16.86 ± 4.15^C^ |
| DMSO 2X | 19.566 ± 5.39^A^ | 26.18 ± 3.57^A^ | 33.4 ± 3.79^A^ | 19.22 ± 3.14^A^ | 24.1 ± 1.55^A^ | 6.772 ± 1.34^AB^ |
| Ferr-1 2X | 15.628 ± 8.24^A^ | 13.8 ± 2.2^D^ | 24.18 ± 2.09^D^ | 12.94 ± 1.54^D^ | 19.78 ± 2.43^D^ | 5.074 ± 0.76^A^ |
| NAC 2X | 16.044 ± 5.44^A^ | 14.812 ± 2.18^D^ | 31.38 ± 4.81^A^ | 13.14 ± 2.05^D^ | 15.264 ± 0.97^D^ | 4.72 ± 1.14^A^ |
| p-value | 0.0001 | 0.0001 | 0.0001 | 0.0001 | 0.0001 | 0.0001 |

Spectral flow cytometry results for ferroptosis modulation on Day 3 were analyzed using one-way ANOVA followed by Tukey’s post hoc test. Values are presented as mean ± SEM (n = 5). Within each column, different superscript letters indicate significant differences between groups (p < 0.05), whereas shared letters denote no significant difference (p ≥ 0.05).

**Supplementary Table S5. Ferroptosis Signatures in Ferrous Iron-Accumulating (Labile Fe²⁺) Sperm Populations (FO⁺).**

| **Treatment** | **MCB-** | **PI+** | **Bodipy C11-/DCFDA+** | **Bodipy C11+ / DCFDA+** | **MTDR-** |
| --- | --- | --- | --- | --- | --- |
| Sample Only | 76.53 ± 6.871829^A^ | 8.604 ± 1.61883^A^ | 6.78 ± 2.34555^A^ | 84 ± 4.14178^AB^ | 40.215 ± 2.156^A^ |
| DMSO 1X | 73.536 ± 7.431951^A^ | 7.236 ± 1.72781^AB^ | 6.7156 ± 2.49623^A^ | 94.036 ± 3.603^A^ | 48.266 ± 3.57648^AB^ |
| Erastin 1X | 75.184 ± 6.529129^A^ | 11.784 ± 2.50543^AC^ | 6.735 ± 2.82204^A^ | 86.192 ± 3.34683^B^ | 57.96 ± 5.46238^B^ |
| RSL-3 1X | 89.938 ± 10.24601^B^ | 16.096 ± 3.5^C^ | 15.6858 ± 4.15165^B^ | 85.1418 ± 3.82298^B^ | 59.494 ± 5.64004^B^ |
| DMSO 2X | 73.972 ± 6.010791^A^ | 8.978 ± 1.6995^A^ | 5.301 ± 2.68882^A^ | 90.9552 ± 5.5215^AB^ | 47.83 ± 3.54137^AB^ |
| Ferr-1 2X | 57.19 ± 3.50734^C^ | 5.084 ± 1.20424^B^ | 1.696 ± 1.0417^C^ | 68.968 ± 7.28021^C^ | 33.574 ± 3.95462^C^ |
| NAC 2X | 60.2564 ± 3.549286^C^ | 6.04 ± 1.27683^B^ | 1.734 ± 0.77432^C^ | 89.1482 ± 5.4268^AB^ | 36.664 ± 3.429547^C^ |
| p-value | 0.009 | 0.028 | 0.002 | 0.0001 | 0.0001 |

Spectral flow cytometry results for ferroptosis modulation in FO-positive sperm cells on Day 3 were analyzed using one-way ANOVA followed by Tukey’s post hoc test. Values are presented as mean ± SEM (n = 5). Within each column, different superscript letters indicate significant differences between groups (p < 0.05), whereas shared letters denote no significant difference (p ≥ 0.05).

**Supplementary Table S6. Biomarker progression in untreated samples across storage days.**

| **Day** | **FO+** | **MCB-** | **DCFDA+** | **BODIPY C11+** | **MTDR-** | **PI+** |
| --- | --- | --- | --- | --- | --- | --- |
| 0 | 3.632 ± 1.0399^A^ | 5.086 ± 0.81808^A^ | 17.492 ± 4.43509^A^ | 6.42 ± 2.759744^A^ | 8.734 ± 1.415399^A^ | 2.156 ± 0.200314^A^ |
| 1 | 16.86 ± 4.0107^B^ | 10.08 ± 1.19933^B^ | 22.3 ± 1.46731^A^ | 8.39 ± 1.249839^A^ | 9.52 ± 1.621169^A^ | 2.286 ± 0.125031^A^ |
| 3 | 18.072± 6.010^B^ | 24.46 ± 3.60951^C^ | 30.24 ± 1.3966^A^ | 19.35 ± 3.294085^B^ | 22.48 ± 1.38686^B^ | 5.78 ± 1.0518^B^ |
| 7 | 33.62 ± 1.16679^C^ | 32.52 ± 5.080787^D^ | 61.62 ± 1.8440^B^ | 33.14 ± 2.27663^C^ | 40.56 ± 5.151465^C^ | 9.864 ± 1.6867^C^ |
| p-value | 0.0001 | 0.0001 | 0.0001 | 0.0001 | 0.0001 | 0.0002 |

Spectral flow cytometry results for ferroptosis modulation in single sperm cells in untreated samples were analyzed using one-way ANOVA followed by Tukey’s post hoc test. Values are presented as mean ± SEM (n = 5). Within each column, different superscript letters indicate significant differences between groups (p < 0.05), whereas shared letters denote no significant difference (p ≥ 0.05)

**Supplementary Table S7. Ferroptosis Biomarker Fluorescence results in FO-positive sperm cells in the untreated control at different timepoints.**

| **Day** | **MCB-** | **PI+** | **Bodipy C11- / DCFDA+** | **Bodipy C11+ / DCFDA+** | **MTDR-** |
| --- | --- | --- | --- | --- | --- |
| 0 | 36.566 ± 4.30155^A^ | 5.48 ± 0.500133^A^ | 66.72 ± 6.3^A^ | 28.982 ± 3.2^A^ | 31.328 ± 3.63869^A^ |
| 1 | 51.794 ± 6.60735^B^ | 2.286 ± 0.556728^B^ | 24.1666 ± 2.9^B^ | 68.762 ± 3.6^B^ | 31.328 ± 4.37086^A^ |
| 3 | 76.53 ± 6.87183^C^ | 8.604 ± 1.61883^C^ | 6.78 ± 2.34555^C^ | 84 ± 4.14178^C^ | 40.215 ± 1.50231^B^ |
| 7 | 91.502 ± 1.98478^D^ | 12.8 ± 2.038143^D^ | 9.6882 ± 2.3^C^ | 86 ± 6.6^C^ | 71.066 ± 10.4495^C^ |
| p-value | 0.0001 | 0.002 | 0.0001 | 0.0001 | 0.0001 |

Spectral flow cytometry results for ferroptosis modulation in FO-positive sperm cells on Day 3 were analyzed using one-way ANOVA followed by Tukey’s post hoc test. Values are presented as mean ± SEM (n = 5). Within each column, different superscript letters indicate significant differences between groups (p < 0.05), whereas shared letters denote no significant difference (p ≥ 0.05).
